# A biophysical elucidation for less toxicity of Agglutinin than Abrin-a from the Seeds of *Abrus Precatorius *in consequence of crystal structure

**DOI:** 10.1186/1423-0127-17-34

**Published:** 2010-04-30

**Authors:** Jack Cheng, Tian-Huey Lu, Chao-Lin Liu, Jung-Yaw Lin

**Affiliations:** 1 Department of Physics, National Tsing Hua University, Hsinchu 30013, Taiwan; 2 Graduate School of Biochemical Engineering, Ming Chi University of Technology, Taishan, Taipei, 24301, Taiwan; 3 Institute of Biochemistry, College of Medicine, National Taiwan University, Taipei 10018, Taiwan

## Abstract

X-ray crystal structure determination of agglutinin from *abrus precatorius *in Taiwan is presented. The crystal structure of agglutinin, a type II ribosome-inactivating protein (RIP) from the seeds of *Abrus precatorius *in Taiwan, has been determined from a novel crystalline form by the molecular replacement method using the coordinates of abrin-a as the template. The structure has space group P4_1_2_1_2 with Z = 8, and been refined at 2.6 Å to R-factor of 20.4%. The root-mean-square deviations of bond lengths and angles from the standard values are 0.009 Å and 1.3°. Primary, secondary, tertiary and quaternary structures of agglutinin have been described and compared with those of abrin-a to a certain extent. In subsequent docking research, we found that Asn200 of abrin-a may form a critical hydrogen bond with G4323 of 28SRNA, while corresponding Pro199 of agglutinin is a kink hydrophobic residue bound with the cleft in a more compact complementary relationship. This may explain the lower toxicity of agglutinin than abrin-a, despite of similarity in secondary structure and the activity cleft of two RIPs.

## Background

Ribosome inactivating proteins (RIPs) are enzymes that can inactivate ribosomes. The molecular mechanism of inhibitory effect on protein synthesis has been shown that RIPs act as a RNA N-glycosidase hydrolyzing the C-N glycosidic bond of the adenosine residue at position 4324 in rat 28S rRNA [1,2]. They can cleave the synthetic RNA structure having a short double-helical stem and a loop containing a centered GAGA sequence, the first A being the cleavage site [3]. The depurination inactivates the ribosomes for binding to elongation factor 2 catalyzing GTP hydrolysis and translocation of peptidyl-tRNA to the P site [4], with a consequence inhibiting the protein synthesis. There are three categories of RIPs according to the physical composition and characteristics. Most commonly RIPs are type I RIPs, only single polypeptide chain proteins composed of the toxophoric A subunit with a molecular mass around 30 kDa [5-8] such as curcin [9] and trichomislin [10]. Some are type II RIPs consisting of two types of polypeptide subunits, A chain of homologous and functionally similar to type I RIPs and B chain with a galactose-specific lectin domain that binds to cell surfaces, such as ricin [11] abrin and abrus agglutinin (AAG) [12]. A chain and B chain are from one gene and link through disulfide bond after post-translation modification [13]. Type III RIPs are derived from inactive pro-protein and activated after proteolysis [14]. The mature type III RIPs are two polypeptide subunits acting as an N-glycosidase jointly.

Various RIPs can be isolated from the same plants [15,16]. Some type II RIPs have been isolated from the beans of the tropical and subtropical leguminous plant *Abrus precatorius*, jequirity. They are lectins and have an inhibitory effect on the growth of experimental animal tumors [17,18]. They can be classified as abrins and AAG by oligomerization. Abrins are potent toxic heterodimeric glycoproteins with an LD50 of 20 μg/kg body weight; while AAG is a relatively less toxic heterotetrameric glycoprotein of which the LD50 is 5 mg/kg body weight [12]. But their therapeutics indexes are similar [18].

The primary structures of abrin-a and AAG were determined [19-21]. AAG had high homology to the extremely toxic ABRa, with 44 (8.0%) similar amino acid residues and 382 (69.8%) invariant amino acid residues. In the A chain of AAG, the 13 amino acid residues with catalytic function among RIPs were completely conserved [21]. The cDNAs of the RIPs isolated from *Abrus precatorius *have been cloned and their A chains were expressed in Escherichia. coli [21-23]. The amino acid residues at proposed active sites and Pro199 of AAG, which corresponding to Asn200 of abrin-a, were analyzed with site-directed mutagenesis for studying the structure and function of these RIPs [21,23,24]. And the results showed that Pro199 in A- (or C-) chain of AAG impair the activity of protein synthesis inhibition because of steric hindrance [21]. According to the biochemical experiments, the mutation of Asn200 on abrin a-chain to Pro200 dramatically decreases the activity than other kind of mutation, including those residues without side-chain, such as Gly [23,24]. These peculiar results motivate us to crystallize AAG, and make comparison with abrin, since both contains almost identical active pocket, and most important of all, different at Asn200 (the corresponding residue is on AAG Pro199). Bagaria et al., [25] reported a 3.5 Å X-ray crystal structure, and proposed the less toxic nature is because of the fewer interactions involved with the substrate adenine.

Bagaria et al., [25] assigned their low resolution of AAG crystal to belong to the space group of P4_2_2_1_2, instead of our present and previous P4_1_2_1_2 [26], to analyze the crystal structure based on a mixture of indigenous and alien data. They crystallized their Indian AAG material in a condition similar to, but different from ours [25,26]. Strange to us, they did not determine their own Indian AAG amino acid sequence, but adopted the Taiwanese primary structure [21,25]. Indian AAG molecular packing may be different from our Taiwanese that could manifest itself some way in different space group. Although they published the controversial paper of 60 kDa structure in advance [25], this detail worthwhile work of more complicated and precise 120 kDa heterotetramer agglutinin structure spurs the continuous study of our last research [26].

## Methods

### Purification

AAG was isolated from the kernels of *Abrus precatorius *seeds by chromatographies on a Sepharose 6B column and a Sephadex G-100 column as described previously [12]. The flow rate of chromatography was 20 ml/hr and protein concentration was determined by the bicinchonic acid method [27]. The kernels of 200 g were soaked in 5% cold acetic acid of 1 L overnight and homogenized. After centrifuging at 10,000 g at 4°C for 15 mins, the supernatant was collected for subsequently subjecting to the ammonium sulfate fraction between 35 and 90 and then centrifuging at 10,000 g at 4°C for 20 mins. The precipitate was collected for dialysis against cold 10 mM sodium phosphate buffer, pH 8 at 4°C. The dialysis buffer was changed every 8 hrs for more than 2 days. The supernatant of dialysate was centrifuged at 17,800 g at 4°C for 20 mins and then applied on a Sepharose 6B affinity column (3.0 × 50 cm) pre-equilibrated and washed with 10 mM sodium phosphate buffer, pH 8. The eluent constiting of abrins and AAG were obtained with the elution buffer, the wash buffer containing 100 mM D-galactose. Then the precipitate was obtained from the eluent subjected to 90% ammonium sulfate and dialyzed and centrifuged as mentioned above. The supernatant was loaded onto gel filtration on Sephadex G-100 column (2.2 × 100 cm) with 10 mM sodium phosphate buffer, pH 8. Two major peaks can be observed and the fractions of AAG, corresponding to the first peak, were pooled and lyophilized.

### Crystallization

The formula for crystallization was described in our previous paper [26]. Crystals suitable for X-ray analysis were obtained by the sitting drop vapor-diffusion method at room temperature (297 (2) K) [28]. 8 μl of protein solution at a concentration of 10 mg/ml prepared from lyophilized protein was mixed with 8 μl of reservoir solution containing PEG 8000; the precipitant condition was 0.1 M Tris pH 7.5 with 6.5% PEG 8000 plus 1% sodium azide and crystals appeared after nearly four months.

### Data Collection

X-ray Data were collected with a crystal of dimensions 0.30 × 0.30 × 0.25 mm that was mounted in a cryo-loop manufactured by Hampton Research. After immersed in the cryo-protectant of 20% glycerol and 80% mother liquor for several seconds, the cryo-loop was mounted on goniometer head inside liquid nitrogen stream at 100 K. X-ray diffraction was measured with CCD (ADSC Quantum-Q4R CCD Area Detector), on 1 D synchrotron radiation X-ray (SPring-8 Taiwan Contract Beam-line BL12B2 of NSRRC). The crystal-to-detector distance was 215 mm. The space group and unit-cell parameters were determined from the well resolved diffraction spots. The data were processed using the programs HKL2000 [29]. The agglutinin crystal belongs to the tetragonal system, with unit-cell parameters a = b = 137.05, c = 214.42 Å, V = 4.0275 × 10^6 ^Å^3^, Z = 8. A 99.1% complete dataset to 2.47 Å resolution of 73,976 unique reflections was collected with averaged R_sym _of 7.2%, averaged χ^2 ^of 1.153, averaged I/σ of 11.89, and redundancy of 4.1.

### Determination of space group and initial phase

The systematic absences, l = 4n + 1, 2, 3 for 00l reflections, and h = 2n + 1 for h00 reflections, indicate that there are two possible space groups, namely P4_1_2_1_2 or P4_3_2_1_2. The ambiguity of space group was solved together with the initial phase problem by molecular replacement method using version 1.1 of CNS program [30] with the coordinates of abrin-a [31] as model. An X-ray diffraction data shell from 4 to 15 Å was used for the calculation of the cross rotation function with CNS program [32]. The highest two were corresponding to a rotation of the model by the rotation angel of θ1 = 37.9E, θ2 = 39.6E, θ3 = 342.1E, and θ1 = 358.1E, θ2 = -0.5E, θ3 = 2.4E in the space group of P4_1_2_1_2. After translation searches with CNS program [33] according to these two rotation angles, the initial model of AB- and CD-chains of agglutinin was established.

### Crystallographic Refinement

Structural refinement were performed in the following iteration steps: rigid body refinement [34], simulated annealing [35] of residue coordinates, group B factor refinement [34], density modification [36], manual manipulation using O program [37], and energy minimization [38]. The crystal data and R factor are listed in Table 1. The final R factor using all reflections in the resolution range 2.6 to 30 Å is 20.4%, while R_free _using randomly selected 10% reflections which were excluded from refinement is 23.6%. The Ramachandran plot including A-, B-, C-, and D-chains is acceptable as shown in Table 1.

**Table 1:** Crystal data and refinement statistics for AAG.

Crystal ID	AAG
Agglutinin A-Chain	Residues 1-250

Agglutinin B-Chain	Residues 5-267

Agglutinin C-Chain	Residues 1-250

Agglutinin D-Chain	Residues 5-267

X-ray wavelength (Å)	1

Crystal system	tetragonal

Space group name	P4_1_2_1_2

Cell length a (Å)	137.050

Cell length b (Å)	137.050

Cell length c (Å)	214.424

Cell volume (Å^3)	4027462.2

Cell formula units Z	16

Cell measurement temperature (K)	100

Crystal shape	octahedron

Crystal color	transparent

Crystal size (mm^3)	0.30 × 0.30 × 0.25

Colvent content (%)	72.33

Matthews coefficient (Å^3/Da)	4.45

Unique reflections	73976

Averaged R_sym (outer sell)	0.0727 (0.3600)

Averaged I/FI (outer sell)	11.9 (1.8)

Completeness (%) (outer sell)	99.1 (98.1)

Redundancy (outer sell)	4.1 (3.6)

Resolution range of collection (Å)	2.47 ~ 30.0

Resolution range of refinement (Å)	2.6 ~ 19.88

R_cryst (outer sell)	0.204 (0.211)

R_free (outer sell)	0.236 (0.256)

No. of protein atoms	8062

No. of water molecules	169

No. of NAG atoms	42

rms deviation from ideal bond length (Å)	0.009

rms deviation from ideal bond angle (º)	1.3

Isotropic thermal factor restraints	rms sigma

Main chain bond (Å^2)	1.87; 1.50

Main chain angle (Å^2)	2.84; 2.00

Side chain bond (Å^2)	2.87; 2.00

Side chain angle (Å^2)	3.90; 2.50

Ramachandran plot [50] (% of residues)	

in the most favored regions (A, B, L)	81.7

in the additionally allowed regions (a, b, l, p)	18.3%

### Docking

The program SPHGEN identifies the active site, and other sites of interest, and generates the sphere centers that fill the site. It has been described in the original paper [39]. The program GRID generates the scoring grids [40,41]. Within the DOCK suite of programs, the program DOCK matches spheres (generated by SPHGEN) with ligand atoms and uses scoring grids (from GRID) to evaluate ligand orientations [38,39]. Program DOCK also minimizes energy based scores [42]. Parameters used in DOCK were modified from the paper of protein docking and complementary principle [43].

The atomic coordinates of the refined agglutinin structure and the reflection data have been deposited with the Protein Data Bank in Japan. The accession numbers for these atomic coordinates are (PDB ID) 2ZR1and (RCSB ID) RCSB028317.

## Results and Discussion

**Figure 1:**
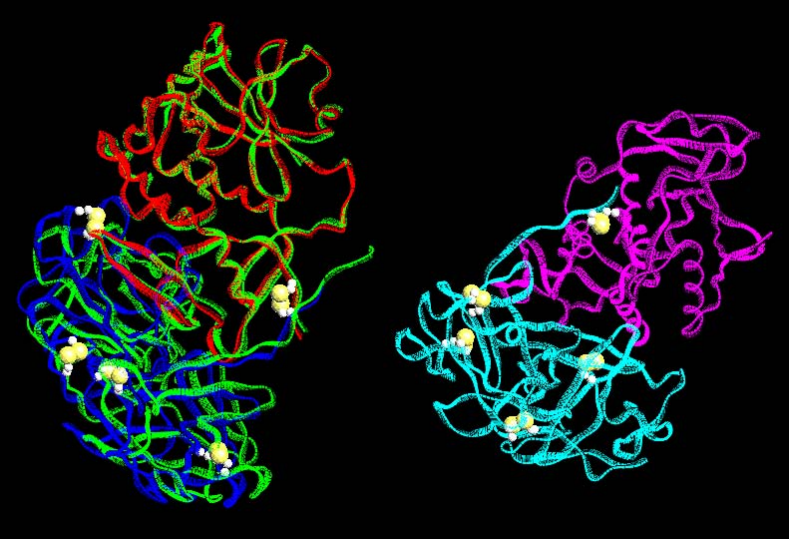
**Comparison of AAG with abrin-a (green) molecule**. The α-carbon backbone of abrin-a AB-chains are superimposed on that of the AAG molecule using least-squares analysis. A P4_1_2_1_2 asymmetric unit of AAG contains an AB-chain and a CD-chain. Disulphide bonds are plotted as big yellow balls. This figure was generated by O program (Jones et al., 1991).

As shown in figure 1, the AAG AB-chains are very similar to the abrin-a molecule, the structure of which has been described in detail [31]. A conserved disulfide bond between Cys246 of A (or C)-chain and Cys8 of B (or D)-chain holds the two chains tightly as shown in figure 1. An asymmetric unit of AAG crystal contains four peptide chains, AB- and non-crystallographical-symmetric related CD-chains, as shown in figure 1. The two heterodimers AB and CD are bonded together through hydrogen bonds by using the water molecules between them as intermediate bridges. They are identical except two N-acetylglucosamines (NAGs) are found in AB-chains, and one in CD-chains. An AAG molecule is a tetramer, consisting of AB (or CD) and symmetry-related A'B' (or C'D').

### Structure of the AAG A(or C)-chain

**Figure 2:**
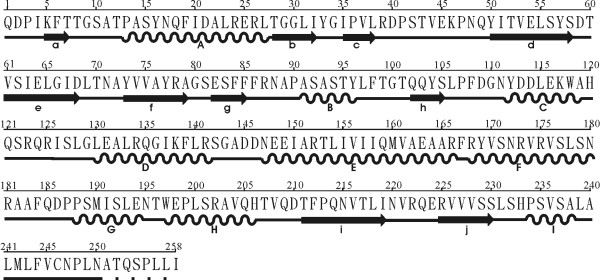
**AAG A (or C)-chain sequence & secondary structures**. The symbol of "arrow" represents a β-strand, "spiral" represents an α-helix, "dot" represents missing residues, and the alphabets a, b, A, etc, denote the corresponding secondary structures in figure 3.

**Figure 3:**
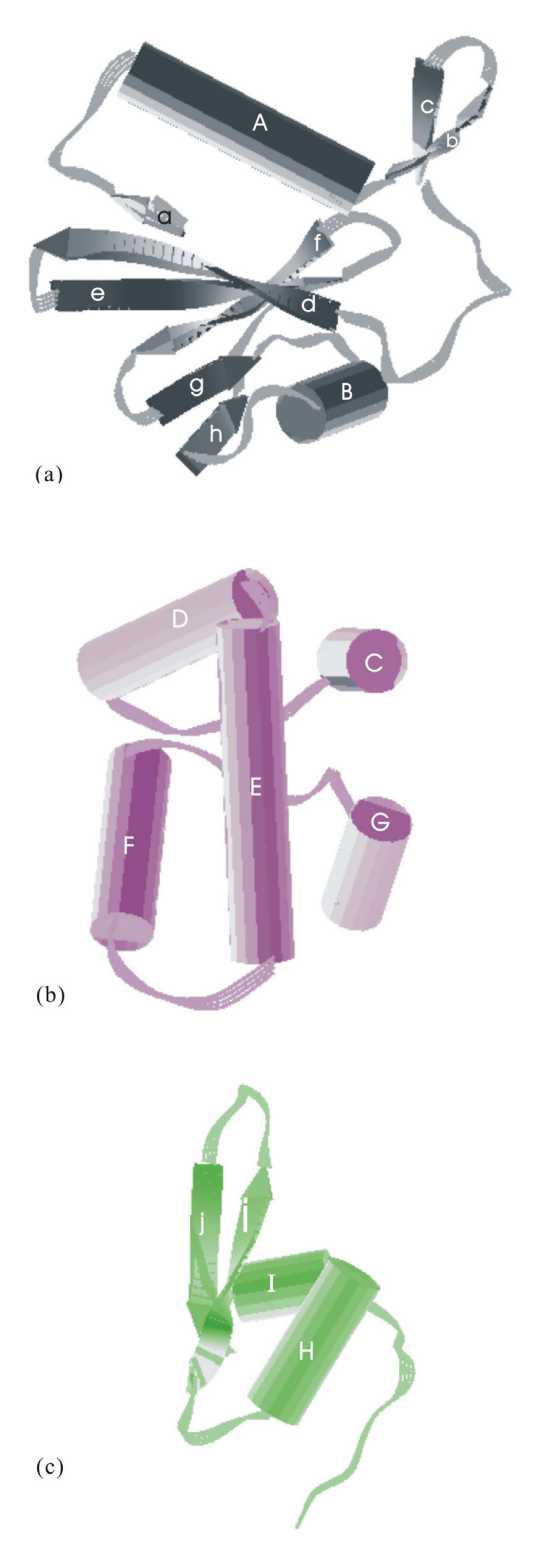
**Three domains of AAG A (or C)-chain: (a) domain γ1, (b) domain γ2, (c) domain γ3**. These figures were generated by O program (Jones et al., 1991).

The AAG A(or C)-chain was divided into three folding domains γ1,γ2, and γ3 by reference to the description of the abrin-a A-chain [31], and to the CATH database [44]. Figure 2 shows the sequence and secondary structures, while figure 3 shows the cartoon of the three domains. Domain γ1 (figure 3(a)), composed of residues 1 to 111, consists of two β-sheets and two α-helices. The former β-sheets include six strands of adefgh (sheet 1) and two strands of bc (sheet 2), while the latter α-helices include helix A of residues 13 to 27, and helix B of residues 91 to 96. The strands and helices alternate in the order aAbcdefgBh. In sheet 1, the first strand, a, of the β-sheet 1 and the last strand, h, lie parallel to the neighboring strands, d and g, respectively. The four central strands of the β-sheet 1, d to g, are anti-parallel. In sheet 2, strands b and c are anti-parallel. The main differences between domains γ1 of AAG and abrin-a occurred in N-terminal. The N-terminal of the AAG A-chain is one residue shorter than that of the abrin-a A-chain and the first five terminal residues are different. Domain γ2, residues 112 to 195, is dominated by five helices (figure 3(b)), C to G. Helix C, composed of residues 112 to 119, D, residues 120 to 141, E, residues 147 to 166, F, residues 168 to 180, and G, residues 188 to 194. Helix C is 3 residues longer than that of abrin-a, due to replacement of Thr114 and Arg118 in abrin-a by Asp113 and Lys117 in AAG. Other secondary structures in domain 2 are almost conserved in abrin-a and AAG. Domain γ3 (figure 3(c)), composed of residues 198 to 250, contains two helices, H, residues 197 to 206 and I, residues 234 to 238, and a β-sheet of two anti-parallel strands, i and j, situated in the order HijI, and a random coil in the C terminal part. The last 8 residues in the C terminal of A-chain are severely disordered, and we could not determine their structures by X-ray diffraction.

### Structure of the AAG B (or D)-chain

**Figure 4:**
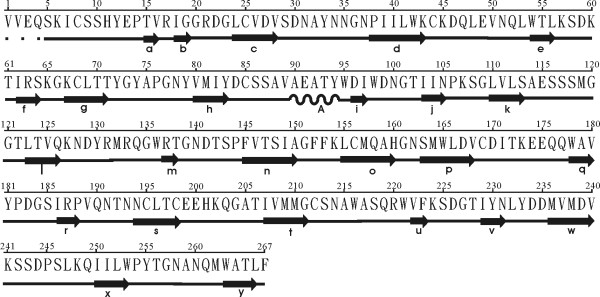
**AAG B (or D)-chain sequence & secondary structures**. The symbol of "arrow" represents a β-strand, "spiral" represents an α-helix, "dot" represents missing residues, and the alphabets a, b, A, etc, denote the corresponding secondary structures in figure 5.

**Figure 5:**
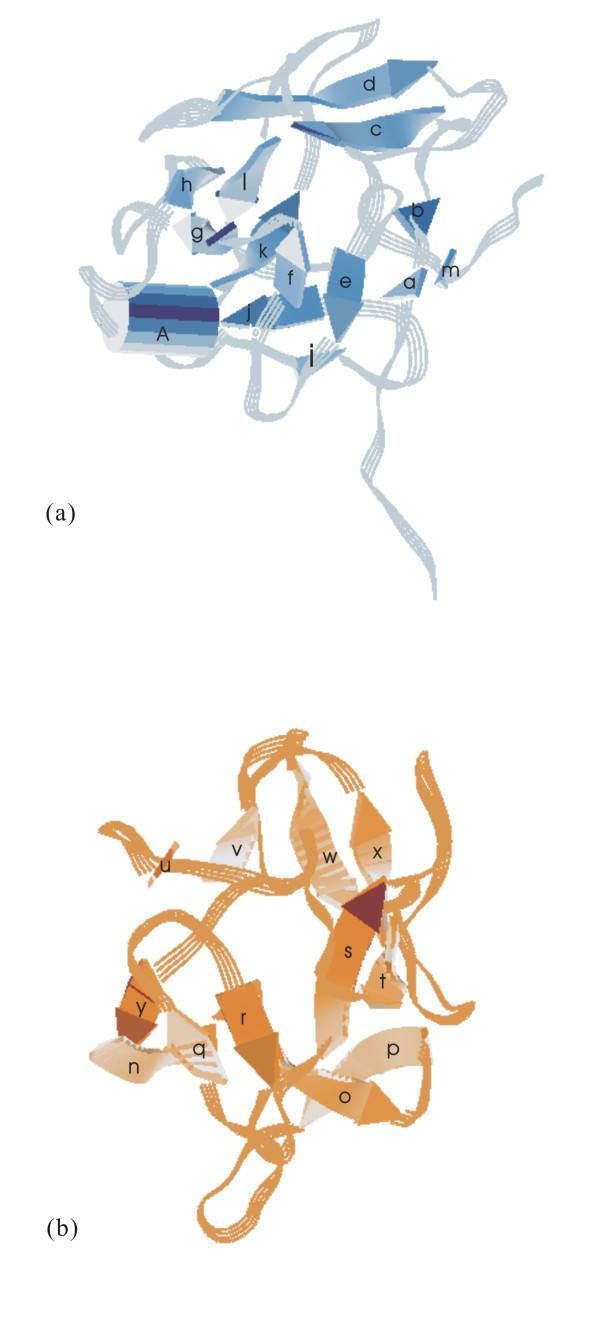
**Two domains of AAG B (or D)-chain: (a) domain δ1, and (b) domain δ2**. These figures were generated by O program (Jones et al., 1991).

The overall folding of the AAG B (or D)-chain and the abrin-a B-chain is very similar, as shown in figure 1, and the disulfide bond connecting A- and B-chains is conserved. The α-carbon traces of their N terminal, residues 1 to 12 differ significantly. The first four residues in the AAG B (or D)-chain are severely disordered, and we could not determine their structures by X-ray diffraction. The AAG B-chain is composed of two homologous domains, δ1 and δ2, mainly formed by β-sheets and loops. Figure 4 shows the sequence and secondary structures, while figure 5 shows the cartoon of the two domains. Domain δ1 (figure 5(a)), composed of residues 5 to 140, consists of five anti-parallel β-sheets, one 4-stranded (of ijkl), one 3-stranded (of aef), and three 2-stranded (strands bm, cd, and gh respectively), and one α-helix of residues 90 to 94. The strands and helices alternate in the order abcdefghAijklm. Domain δ2 (figure 5(b)), composed of residues 141 to 267, consists of four anti-parallel β-sheets, including two 4-stranded (strands ynqr and uvwx respectively), and two 2-stranded (strands op and st) sheets.

**Figure 6:**
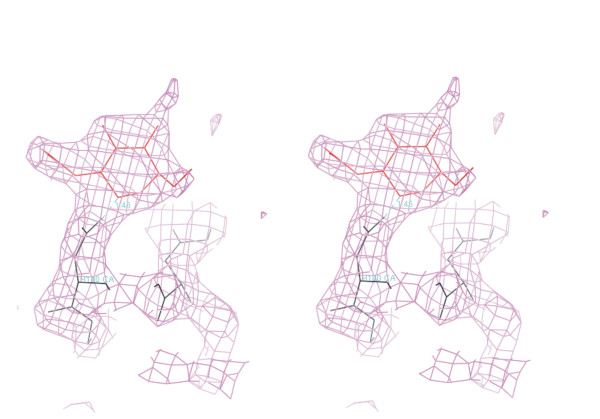
**Electron density of the NAG (red) near B 100Asn using the (2Fo - Fc) map contoured at 2.0 F**. This figure was generated by O program (Jones et al., 1991).

Each domain of δ1 and δ2 contains two intra-domain disulfide bonds (Cys25-Cys44, Cys68-Cys85, Cys156-169, and Cys195-Cys212), which are conserved in abrin-a. Two NAGs are found in B-chain, but only one presents in D chain. The NAGs are bound to B-Asn100 (figure. 6), B-Asn140, and D-Asn140 respectively. The bond length between NAG and Asn140 is 1.45 Å.

### Structure of Active site

**Figure 7:**
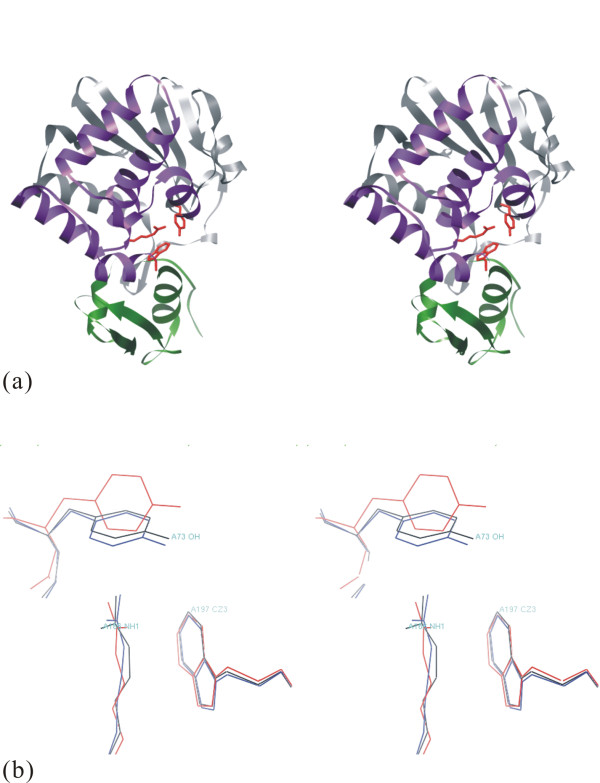
**Three domains of AAG A (or C)-chain are drawn as ribbons**. (a) Gray purple and green indicate domain γ1 and green indicate domain γ1 γ2 γ2 and γ3 respectively. Active site residues are drawn in red. (b) Active Site comparison of abrin-a (red) and γ3 respectively. Active site residues are drawn in red. (b) Active Site comparison of abrin-a (red) AAG A-chain (black) AAG A-chain (black) and AAG C-chain (blue). These figures were generated by O program (Jones et al. and AAG C-chain (blue). These figures were generated by O program (Jones et al. 1991) and UCSF Chimera [32].

The active site is exactly the cleft formed by the intersection of all 3 domains in AAG A (or C)-chain. The location of the active site region of the AAG A (or C)-chain is shown in figure 7(a), and enlarged in figure 7(b). Five invariant residues (Tyr73, Tyr112, Glu163, Arg166 and Trp197) and five conserved residues (Asn71, Arg123, Gln159, Glu194 and Asn195) are located in the active site cleft. The alignment of the amino acid sequences shows that all five invariant residues in the active site of abrin-a are absolutely conserved throughout the wide range of ribosome-inactivating proteins [19,45]. The similarity of active site structures between abrin-a and AAG shows in figure 7(b) that they may work in the same way, but could not explain the less than half biochemical activity of AAG. We try to answer this question by the 28SRNA docking study.

### Quaternary Structure of AAG

**Figure 8:**
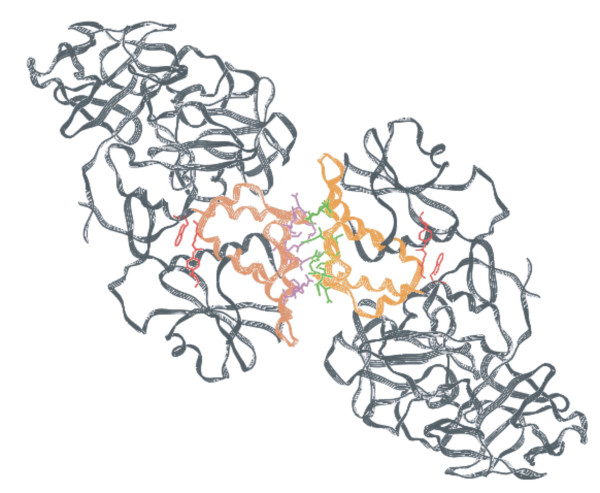
**Ribbon presentation of AAG quaternary structure: Red residues indicate the active site location**. Purple and green residues constitute inter-subunit hydrogen bonds. Domain γ2s are drawn in brown. This figure was generated by O program (Jones et al., 1991).

An AAG molecule is a hetero-tetramer (as shown in figure 8) contains two subunits, ABA'B' (or CDC'D'), stabilized by mainly hydrophilic and little hydrophobic forces. The two subunits are in equivalent positions of the space group P4_1_2_1_2. The transformation from AB to A'B' is (x, y, z) to (1-y, 1-x, 0.5-z), while CD to C'D' is (x, y, z) to (y, x, 1-z). The hydrophilic interaction is dominated by inter-subunit hydrogen bonds, as listed in table 2. These hydrogen bonds belong to residues of domains γ2 and γ2'. Since the γ2 domain is almost made up with α-helices, which hydrophobic side-chains are buried inside, hydrophobic forces contribute little to the stabilization of quaternary structure of AAG. The total buried surface area is 9360 for ABA'B' and 9460 for CDC'D' interfaces. The gain in hydrophobic energy is -68 KCal/Mol for ABA'B' and -72 KCal/Mol for CDC'D'. The buried surface and hydrophobic energy are calculated by Protein interfaces, surfaces and assemblies service PISA at European Bioinformatics Institute http://www.ebi.ac.uk/msd-srv/prot_int/pistart.html[46].

**Table 2:** Hydrogen bonds between inter-subunit with symmetry-related AA' and CC' chains.

Donor	Acceptor	**D....A (**A)	Donor	Acceptor	**D....A **(A)
A Gln 121 NE2	A'Gln121 OE1	2.85	A Gln121 NE2	A'Gln121 OE1	2.85

A Arg 25 NH1	A'Glu148OE2	2.77	A Arg125NH1	A'Glu148OE2	2.77

A Leu 130 N	A'Glu131OE2	3.15	A Leu 130 N	A'Glu131OE2	3.15

A Arg 134 NH2	A'Asn 180 O	2.86	A Arg 134 NH2	A'Asn 180 O	2.86

A Arg 134 NE	A'Asn 181 O	3.06	A Arg 134 NE	A'Asn 181 O	3.06

A Gln 135 NE2	A'Ser 127 OG	3.07	A Gln 135 NE2	A'Ser 127 OG	3.07

C Gln 121 NE2	C'Ser 145 O	2.75	C Gln 121 NE2	C'Ser 145 O	2.75

C Ser 127 OG	C'Gly 143 N	2.95	C Ser 127 OG	C'Gly 143 N	2.95

C Arg 134 NR	C'Tyr57 OH	2.64	C' Arg 134 NR	C Tyr57 OH	2.64

C Gln 135 NE2	C'Gln135 O	2.94	C' Gln 135 NE2	C Gln135 O	2.94

C Ser 145 N	C'Gln121OE1	2.59	C' Ser 145 N	C Gln121 O	2.59

### Docking of 28SRNA to AAG and Abrin-a

**Figure 9:**
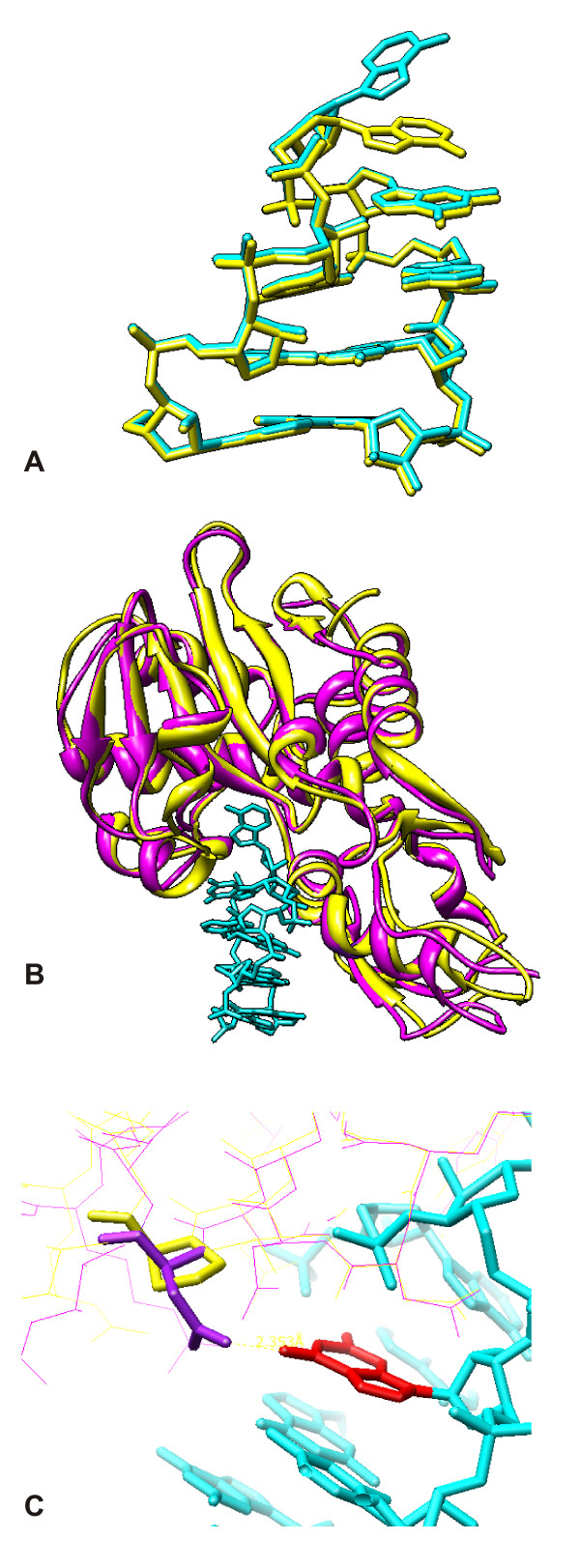
**(a) The docking model (blue) and the original 28SRNA (yellow)**. A4324 was artificially manipulated. (b) The docking of 28SRNA (blue) on abrin-a A-chain (purple) and AAG A-chain (yellow). (c) G4323 (red) of 28SRNA docking model (blue), is hydrogen-bonded with Asn2000 of abrin-a A-chain (purple), but has no interaction with corresponding Pro199 of AAG A-chain (yellow).

As pointed out in the mutagenesis study [21], Pro199 in A- (or C-) chain of AAG impair the activity of protein synthesis inhibition. Bagaria et al., [25] suggested that the less toxic nature is because of the fewer interactions involved with the substrate adenine. From our docking study, we found Asn200 of abrin-a may form a critical hydrogen bond with G4323 of 28SRNA, while corresponding Pro199 of agglutinin is a non-extended residue bound with the cleft in a more compact complementary relationship as shown in figure 9(c). This may explain the lower toxicity of agglutinin, despite of similarity in secondary structure and the activity cleft of two RIPs. The docking model of 28SRNA was artificially deformed at the ribose sugar of A4324 from the X-ray crystal structure 430D [47], as shown in figure 9(a), so that A4324 of 28SRNA can overlap with the adenine of the ricin-adenine complex, 1IFS [48]. Figure 9(b) shows deformed model fit well in the active clefts of both abrin-a and AAG. Figures 9(a), 9(b) and 9(c) were generated with UCSF Chimera [49].

We want to explore whether the difference in the critical residue, bring any change in structure. However, we could not observe significant main-chain distortion due to the difference in the 200 residue on higher resolution structure. Hence, the reason for the lower toxicity of agglutinin than abrin-a might be due to the deformation from inactive to active state of abrin depends on the meta-stable huge helix, composed of helix h and helix g. The mutation of Asn200 to Pro200 destroys the mechanism.

## Conclusion

We have successfully solved the structure of 120 kDa heterotetramer agglutinin AB and CD chains, and reduced the R-factor to 20.4% at 2.6 Å resolution data. Ten disulfide bonds, three N-acetylglucosamines, and 169 water molecules were found in the successive (2Fo-Fc) map. 22 hydrogen bonds between A (or C)-chain and symmetry-related A' (or C') were found. Water molecules were not found in the Bagaria's paper and no subsequent hydrogen bond lengths were listed based on their lower resolution structure [25]. Docking study revealed that due to Pro199, agglutinin is unable to form a critical hydrogen bond with G4323 of 28SRNA, which is found in the docking result of abrin-a. This may explain the lower toxicity of agglutinin than abrin-a, despite of similarity in secondary structure and the activity cleft of two RIPs.

## Abbreviations

RIP: ribosome-inactivating protein; AAG: agglutinin; NAG: N-acetylglucosamine; Fo: observed structure factor; Fc: calculated structure factor; PDB: protein data bank.

## Competing interests

The authors declare that they have no competing interests.

## Authors' contributions

JC collected the X-ray diffraction data, analyzed the crystal structure and prepared the initial manuscript. LTH set up the laboratory of the crystal structure determination, screened the crystallization conditions and got the right one, and advised such studies. LJY and LCL purified the material of the agglutinin.

## References

[B1] Endo Y Misui K Motizuki K Tsurugi K The mechanism of action of ricin and related toxic lectins on eukaryotic ribosomes. The site and the characteristics of the modification in 28 S ribosomal RNA caused by the toxins J Biol Chem 1987 262 5908 5912 3571242

[B2] Endo Y Tsurugi K RNA N-glycosidase activity of ricin A-chain Mechanism of action of the toxic lectin ricin on eukaryotic ribosomes J Biol Chem 1987 262 8128 8130 3036799

[B3] Endo Y Gluck A Wool IG Ribosomal RNA identity elements for ricin A-chain recognition and catalysis J Mol Biol 1991 221 193 207 10.1016/0022-2836(91)80214-F 1920404

[B4] Jimenez A Vazquez DC Plant and Fungal Protein and Glycoprotein Toxins Inhibiting Eukaryote Protein Synthesis Annu Rev Microbiol 1985 39 649 672 10.1146/annurev.mi.39.100185.003245 3904615

[B5] Barbieri L Stirpe F Ribosome-inactivating proteins from plants: properties and possible uses Cancer Surv 1982 1 489 520

[B6] Stirpe F Barbieri L Battelli MG Soria M Lappi DA Ribosome-inactivating proteins leads to increased fungal protection in transgenic tobacco plants Bio-Technology 1992 10 405 412 1368484 10.1038/nbt0492-405

[B7] Hartley MR Lord JM Cytotoxic ribosome-inactivating lectins from plants Biochim Biophys Acta 2004 1701 1 14 15450171 10.1016/j.bbapap.2004.06.004

[B8] Olsnes S The history of ricin abrin and related toxins Toxicon 2004 44 361 370 10.1016/j.toxicon.2004.05.003 15302520

[B9] Lin J Chen Y Xu Y Yan F Tang L Chen F Cloning and expression of curcin a ribosome inactivating protein from the seeds of jatropha curcas Acta Botanica Sinica 2003 45 858 863

[B10] Mi SL An CC Wang Y Chen JY Che NY Gao Y Chen ZL Trichomislin a novel ribosome-inactivating protein a novel ribosome-inactivating protein induces apoptosis that involves mitochondria and caspase-3 Archives of Biochemistry and Biophysics 2005 434 258 265 10.1016/j.abb.2004.11.009 15639225

[B11] Olsnes S Phil A Different biological properties of the two constituent peptide chains of ricin a toxic protein inhibiting protein synthesis Biochemistry 1973 12 3121 3126 10.1021/bi00740a028 4730499

[B12] Lin JY Lee TC Hsu ST Tung TC Isolation of four isotoxic proteins and one agglutinin from jequiriti bean (Abrus precatorius) Toxicon 1981 19 41 51 10.1016/0041-0101(81)90116-1 7222088

[B13] Lord JM Synthesis and intracellular transport of lectin and storage protein precursors in endosperm from castor bean Eur J Biochem 1985 146 403 409 10.1111/j.1432-1033.1985.tb08666.x 3967663

[B14] Mundy J Leah R Boston R Endo Y Stirpe F Genes encoding ribosome-inactivating proteins Plant Mol Biol Rep 1994 12 60 62 10.1007/BF02671573

[B15] Leah R Tommerup H Svendsen I Mundy J Biochemical and molecular characterization of three barley seed proteins with antifungal properties J Biol Chem 1991 266 1564 1573 1899089

[B16] Desvoyes B Poyet JL Schlick JL Adami P Jouvenot M Dulieu P Identification of a biological inactive complex form of pokeweed antiviral protein FEBS Lett 1997 410 303 308 10.1016/S0014-5793(97)00648-0 9237651

[B17] Olsnes S Phil A Cuatrecasas P In Receptors and Recognition Series: The Specificity and Action of Animal Bacterial and Plant Toxins 1982 Chapman and Hall, London 31 131

[B18] Lin JY Li JS Tung TC Lectin Derivatives of Methotrexate and Chlorambucil as Chemotherapeutic Agents J Natl Cancer Inst 1981 66 523 528 6937708

[B19] Funatsu G Taguchi Y Kamenosno M Yanaka M The complete amino acid sequence of the A-chain of abrin-a a toxic protein from the seeds of Abrus precatorius Agric Biol Chem 1988 52 1095 1097

[B20] Chen YL Chow LP Tsugita A Lin JY The complete primary structure of abrin-a B chain FEBS Lett 1992 309 115 118 10.1016/0014-5793(92)81076-X 1505674

[B21] Liu CL Tsai CC Lin SC Wang LI Hsu CI Hwang MJ Lin JY Primary Structure and Function Analysis of the Abrus precatorius Agglutinin A Chain by Site-directed Mutagenesis J Biol Chem 2000 275 1897 1901 10.1074/jbc.275.3.1897 10636890

[B22] Hung CH Lee MC Lee TC Lin JY Primary Structure of Three Distinct Isoabrins Determined by cDNA Sequencing: Conservation and Significance J Mol Biol 1993 229 263 267 10.1006/jmbi.1993.1029 8421313

[B23] Hung CH Lee MC Chen JK Lin JY Cloning and expression of three abrin A-chains and their mutants derived by site-specific mutagenesisin Escherichia coli Eur J Biochem 1994 219 83 87 10.1111/j.1432-1033.1994.tb19917.x 8307038

[B24] Chen JK Hung CH Liaw YC Lin JY Identification of amino acid residues of abrin-a A chain is essential for catalysis and reassociation with abrin-a B chain by site-directed mutagenesis Protein Engineering 1997 10 827 833 10.1093/protein/10.7.827 9342148

[B25] Bagaria A Surendranath K Ramagopal UA Ramakumar S Karande AA Structure-Function Analysis and Insights into the Reduced Toxicity of Abrus precatorius Agglutinin I in Relation to Abrin J Biol Chem 2006 281 34465 34474 10.1074/jbc.M601777200 16772301

[B26] Panneerselvam K Lin SC Liu CL Liaw YC Lin JY Lu TH Crystallization of agglutinin from the seeds of Abrus precatorius Acta Cryst 2000 D56 898 899 10.1107/s0907444900005047 10930837

[B27] Smith PK Krohn RI Hermanson GT Mallia AK Gartner FH Provenzano MD Fujimoto EK Goeke NM Olson BJ Klenk DC Measurement of protein using bicinchoninic acid Anal Biochem 1985 150 76 85 10.1016/0003-2697(85)90442-7 3843705

[B28] McPherson A Preparation and Analysis of Protein Crystals 1982 John Wiley & Sons John Wiley & Sons New York USA 94 96 115

[B29] Otwinowski Z Minor W Processing of X-ray Diffraction Data Collected in Oscillation Mode Methods in Enzymology. Macromolecular Crystallography part A 1997 276 307 326 full_text 10.1016/S0076-6879(97)76066-X 27754618

[B30] Brunger AT Adams PD Clore GM Delano WL Gros P Grosse-Kunstleve RW Jiang JS Kuszewski J Nilges M Pannu NS Read RJ Rice LM Simonson T Warren GL Crystallography & NMR system: A new software system for macromolecular structure determination Acta Cryst 1998 D54 905 921 10.1107/s0907444998003254 9757107

[B31] Tahirov TH Lu TH Liaw YC Chen YL Lin JY Crystal Structure of Abrin-a at 2.14 D J Mol Biol 1995 250 354 367 10.1006/jmbi.1995.0382 7608980

[B32] DeLano WL Brunger AT The Direct Rotation Function: Rotational Patterson Correlation Search Applied to Molecular Replacement Acta Cryst 1995 D51 740 748 10.1107/S0907444995001284 15299804

[B33] Brunger AT Extension of molecular replacement: A new search strategy based on Patterson correlation refinement Acta Cryst 1990 A46 46 57

[B34] Brunger AT The Free R Value: a Novel Statistical Quantity for Assessing the Accuracy of Crystal Structures Nature 1992 355 472 474 10.1038/355472a0 18481394

[B35] Brunger AT Krukowski A Erickson J Slow-Cooling Protocols for Crystallographic Refinement by Simulated Annealing Acta Cryst 1990 A46 585 593 10.1107/s0108767390002355 2206482

[B36] Abrahams JP Leslie AG W Methods used in the structure determination of bovine mitochondrial F1 ATPase Acta Cryst 1996 D52 30 42 10.1107/S0907444995008754 15299723

[B37] Jones TA Zou JY Cowan SW Kjeldgaard M Improved methods for the building of protein models in electron density maps and the location of errors in these models Acta Cryst 1991 A47 110 119 10.1107/s0108767390010224 2025413

[B38] Adams PD Pannu NS Read RJ Brunger AT Cross-validated Maximum Likelihood Enhances Crystallographic Simulated Annealing Refinement Proc Natl Acad Sci USA 1997 94 5018 5023 10.1073/pnas.94.10.5018 9144182 PMC24623

[B39] Kuntz ID Blaney JM Oatley SJ Langridge R Ferrin TE A geometric approach to macromolecule-ligand interactions J Mol Biol 1982 161 269 288 10.1016/0022-2836(82)90153-X 7154081

[B40] Shoichet BK Bodian DL Kuntz ID Molecular docking using shape descriptors J Comp Chem 1992 13 380 397 10.1002/jcc.540130311

[B41] Meng EC Shoichet BK Kuntz ID Automated docking with grid-based energy evaluation J Comp Chem 1992 13 505 524 10.1002/jcc.540130412

[B42] Meng EC Gschwend DA Blaney JM Kuntz ID Orientational sampling and rigid-body minimization in molecular docking Proteins 1993 17 266 278 10.1002/prot.340170305 8272425

[B43] Shoichet BK Kuntz ID Protein docking and complementarity J Mol Biol 1991 221 327 346 10.1016/0022-2836(91)80222-G 1920412

[B44] Pearl F Todd A Sillitoe I Dibley M Redfern O Lewis T Bennett C Marsden R Grant A Lee D Akpor A Maibaum M Harrison A Dallman T Reeves G Diboun I Addou S Lise S Johnston C Sillero A Thornton J Orengo C The CATH Domain Structure Database and related resources Gene3D and DHS provide comprehensive domain family information for genome analysis Nucl Acids Res 2005 33 Database D247 D251 15608188 10.1093/nar/gki024 PMC539978

[B45] Rutenber E Robertus JD Structure of ricin B-chain at 2.5 Å resolution Proteins Struct Func Genet 1991 10 260 269 10.1002/prot.340100310 1881882

[B46] Krissinel E Henrick K Inference of macromolecular assemblies from crystalline state J Mol Biol 2007 372 774 797 10.1016/j.jmb.2007.05.022 17681537

[B47] Correll CC Munishkin A Chan YL Ren Z Wool IG Steitz TA Crystal structure of the ribosomal RNA domain essential for binding elongation factors Proc Natl Acad Sci USA 1998 95 13436 13441 10.1073/pnas.95.23.13436 9811818 PMC24837

[B48] Weston SA Tucker AD Thatcher DR Derbyshire DJ Pauptit RA X-ray structure of recombinant ricin A-chain at 1.8 Å resolution J Mol Biol 1994 244 410 422 10.1006/jmbi.1994.1739 7990130

[B49] Pettersen EF Goddard TD Huang CC Couch GS Greenblatt DM Meng EC Ferrin TE UCSF Chimera - A Visualization System for Exploratory Research and Analysis J Comput Chem 2004 25 1605 1612 10.1002/jcc.20084 15264254

[B50] Laskowski RA MacArthur MW Moss DS Thornton JM PROCHECK: a program to check the stereochemical quality of protein structures J App Cryst 1993 26 283 291 10.1107/S0021889892009944

